# Autonomous Docking Based on Infrared System for Electric Vehicle Charging in Urban Areas

**DOI:** 10.3390/s130202645

**Published:** 2013-02-21

**Authors:** Joshué Pérez, Fawzi Nashashibi, Benjamin Lefaudeux, Paulo Resende, Evangeline Pollard

**Affiliations:** IMARA Team at INRIA Research Center, Domaine de Voluceau-Rocquencourt, BP 105, 78153 Le Chesnay, France; E-Mails: fawzi.nashashibi@inria.fr (F.N.); benjamin.lefaudeux@inria.fr (B.L.); paulo.lopes_resende@inria.fr (P.R.); evangeline.pollard@inria.fr (E.P.)

**Keywords:** autonomous parking, electric vehicle, vision systems, docking system, lateral control

## Abstract

Electric vehicles are progressively introduced in urban areas, because of their ability to reduce air pollution, fuel consumption and noise nuisance. Nowadays, some big cities are launching the first electric car-sharing projects to clear traffic jams and enhance urban mobility, as an alternative to the classic public transportation systems. However, there are still some problems to be solved related to energy storage, electric charging and autonomy. In this paper, we present an autonomous docking system for electric vehicles recharging based on an embarked infrared camera performing infrared beacons detection installed in the infrastructure. A visual servoing system coupled with an automatic controller allows the vehicle to dock accurately to the recharging booth in a street parking area. The results show good behavior of the implemented system, which is currently deployed as a real prototype system in the city of Paris.

## 1. Introduction

Today, a wide variety of Advanced Driver Assistance Systems (ADAS) are available in conventional vehicles. These systems allow multiple improvements in driving assistance and some partial control such as: blind angle detection systems [1], lane departure warning [2], speed limit warning [3], pedestrian collision avoidance [4] and parking assistance [5] (among others). Nevertheless, some other intelligent transportation systems (ITS) topics are improved for research groups around the world. The European Union, specifically the Directorate-General for Mobility and Transport of the European Commission (EC), develops transport policies by integrating citizen needs, environmental policy and competitiveness [6].

One of the main objectives of the EC is to decrease the use of gas-propelled vehicles in 2050, reducing transport sector emissions of greenhouse gas (GHG) by about 60%. Therefore, electric vehicles (EV) will improve urban transportation, because of their efficiency and the absence of CO_2_ gas emissions and noise. In fact, some fully automated electric vehicles are already in use in airports, private tracks and pedestrian zones in urban areas [7,8]. However, the market penetration of EV depends on the improvement of electric vehicle batteries, in terms of battery costs, operational autonomy and the distribution of charging point availability in the cities.

With the growth of the EV industry, more charging points will appear at motorway service stations and in major cities. This year, in the United States, there are more than eight thousand public charging stations [9]. However, there are still some things lacking in this solution due to the slow charging times and parking problems. Some authors have applied inductive power transfer (IPT) techniques for EV recharging [10]. Although this system offers a safe, convenient and reliable solution, its implementation depends on the performance of the power pads, and this technology is unavailable for all types of EV's. Other works are focusing on the fast-charging electric issues, performing simulations of a recharging station with different platforms [11]. These first results suggest a quick implementation of charging stations for EVs in urban and inter-urban scenarios.

In the last few years, autonomous vehicles, chiefly using EV, have been gradually improved in terms of safety and redundancy. Cybercars are a good example of this evolution, since they allow fully autonomous driving capabilities for specific scenarios in order to provide an on-demand door-to-door service [7]. These vehicles use a GPS sensor for positioning and wireless communications for interaction with other vehicles and the infrastructure [12]. The IMARA group of INRIA (National Institute for Research in Computer Science and Control, France) is working on the development of perception and control strategies for Cybercars [8,13].

Other works propose the control of autonomous EV with mathematical modeling of the motion dynamics and drivability control to optimize the operating freedom of two power trains in hybrid electric vehicles [14]. In [15], a real vehicle modified with a steer-by-wire system and global positioning system (GPS) for localization is proposed. Moreover, artificial intelligence (AI) techniques, such as fuzzy logic [12] and neural-networks (NN) [16], have been used to control real vehicles in urban and highway scenarios, based on human experiences and cooperative GPS and inertial systems [17].

About the localization problem in autonomous vehicles, the limitations of the GPS systems, caused by GPS outages and the interferences in urban and indoor scenarios, are widely known (because of buildings, trees, bridges and parking, among others) [18]. For this reason, other approaches focusing on perception solutions for localization and environment mapping have been suggested, such as SLAM and SLAMMOT, among others [19,20]. A survey of the most important algorithms for autonomous vehicles, based on vision and laser, proposed in the last decade, has been presented in [21]. They claim that, even if many navigation systems are based on heterogeneous sensor data fusion, the most robust algorithms are based on visual target tracking, since the position and velocity of the vehicle and the target relative position can be established by processing the image streams of the cameras.

Autonomous charging is a well identified issue for electric autonomous systems. This problem has been historically addressed in robotics [22], and several approaches were proposed based on a wide range of techniques, such as range lights [23] or vision and artificial landmarks [24]. It is really close to the problem of docking autonomous underwater vehicles (AUVs) for charging purposes [25]. Concerning autonomous urban vehicles, systems using inductive charging were already proposed [26], but are not really energy efficient, even if they are easy to handle. Systems that consider docking for charging electric vehicles require highly accurate localization and control (a few centimeters), uncommonly treated in the literature [27,28]. The aim of the research is to design and develop a control system for automatic recharge docking for EVs in urban parking areas. The vehicle is equipped with an infrared camera, able to detect infrared diodes placed in the infrastructure. These diodes are used as landmarks in order to provide a highly accurate position and velocity to the control stage. The camera is placed behind the rear-view mirror (looking ahead), and the vehicle is an electric Citroën C1, instrumented to enable autonomous driving.

This paper is organized as follows: a description of the system architecture and of the AMARE project objectives are provided in Section 2. The perception algorithms, signal filtering and control stages, followed by an explanation on the control strategies used in the lateral control law, are explained in Section 3. Experimental demonstrations and results obtained with the real facilities are described in Section 4. The paper ends with conclusions and future work in Section 5.

## 2. System Architecture

The automatic docking, recharging, billing and payment system proposed in this paper is composed of three main elements: an automated vehicle, a docking and recharging station and a wireless communication system.

**Figure 1.**
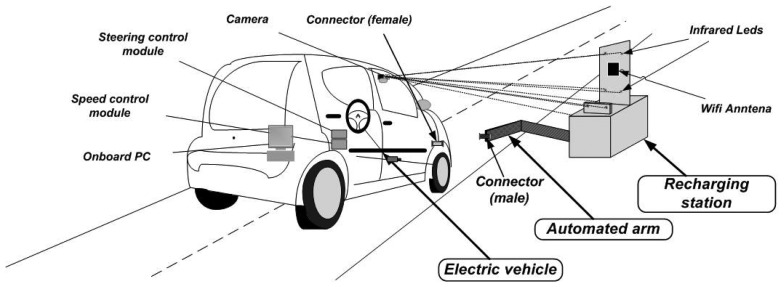
Elements of the system and docking maneuver of the AMARE project.

Once the vehicle is properly parked by the driver a few meters from the station, the perception system identifies the infrared LEDs placed in the recharging station, and then, the connection procedure is initiated by the vehicle. The first connection is performed by wireless communications. The vehicle sends to the station its intention to park and to recharge its batteries. Once accepted by the station, the vehicle autonomously docks with the station, and the recharging starts without any human intervention (Figure 1). When the vehicle intends to leave the station, billing is calculated given the energy consumed by the vehicle and the total parking time. The payment of the charge can be performed via a contactless payment system or sent to the driver's billing address for an *a posteriori* payment.

### 2.1. Automated Vehicle

**Figure 2.**
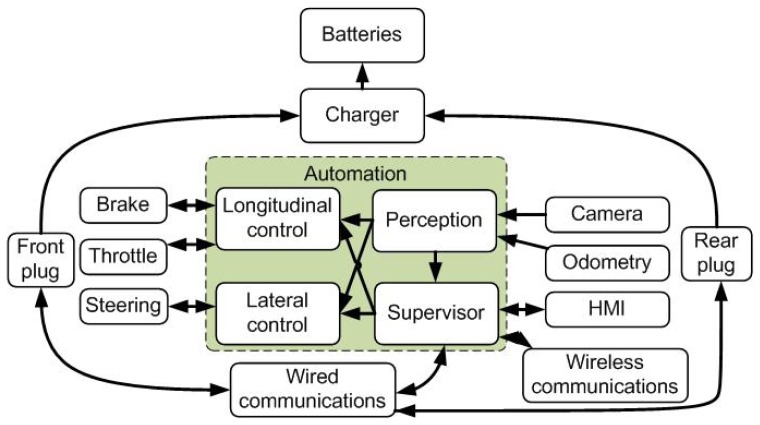
Automated vehicle on the AMARE project.

The vehicle used is an electrified Citroёn C1 instrumented for autonomous driving. Figure 2 shows the different components used in the autonomous vehicle. The vehicle is equipped with two docking plugs—front and back—for battery charging and wired communications. This design allows the connection of several vehicles in series to a unique recharging station, reducing the number of recharging stations needed. In the meantime, the physical link between the vehicles can be used to displace all the vehicles in a platoon configuration with a unique driver, thus facilitating the redistribution of vehicles between stations [29].

A wireless communication link is established before the docking procedure with the recharging station. The automated vehicle uses the information from the installed infrared camera and the odometry to guide the vehicle into its final parking position or docking spot. The perception system (Section 3.1) starts by estimating the pose of the vehicle relative to the pattern of infrared LEDs in the recharging station.

The control of the vehicle and driving task is supported by the on-board automation system until the vehicle reaches its docking spot. The throttle and brake pedals, with integrated potentiometers, are commanded by the longitudinal controller, and the electric power-assisted steering actuator is commanded by the lateral controller (Section 3.2).

Information about the automatic docking procedures are provided to the driver via the on-board HMI (Human Machine Interface) and stored in a remote server. Once the docking is established and the vehicle is plugged into the automated arm, a wired connection is established between the vehicle and the station. At the same time, the recharging of the vehicle batteries starts and the consumed energy is registered. This information is used later by the billing process together with the parking time costs.

### 2.2. Recharging and Docking Station

**Figure 3.**
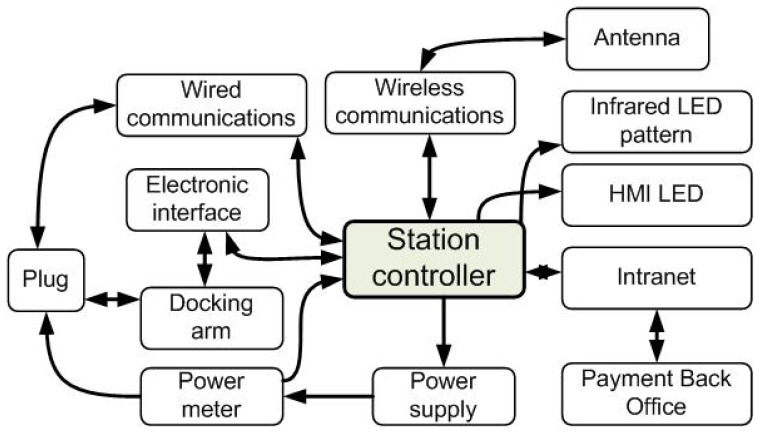
Recharging and docking station on the AMARE project.

The station, equipped with a docking arm, is used to charge up to five vehicles in series. The communication with the vehicle and prior plug-in connection is done via wireless WiFi communications. The station allows/rejects connection requests from the vehicles that want to dock and recharge. The status of the station can easily be accessed by the lights' interface information (in the station): green: the station is available; yellow blinking: the docking arm is being deployed; and red: the station is occupied. Once the plug is connected to a vehicle, the power supply is activated and the energy consumption is registered (Figure 3). An infrared LED pattern installed in the station is detected by the vehicle on-board camera in order to determine its relative position. The station controller manages the electronic interface that controls the docking arm, the power supply and the infrared LED pattern. The controller is connected directly to a payment back office through a local network (intranet) and handles the communications with the vehicle.

### 2.3. Communication System

The action coordination between the vehicle automation (supervisor) and the station controller are performed via an IPv6 wireless link based on embedded Linux boxes (4G Cubes) [8]. This communication system is a Vehicle Web Service Communication Framework (VWSCF) that handles service discovery, exposition and fetching of data through the network. For practical reasons, the payment procedure is performed via a different wireless connection using a standard highway contactless payment system. Once the vehicle is plugged into the docking arm, a wired connection is established, and diagnostics data are exchanged between the vehicle and the station.

## 3. Onboard Algorithms: Autonomous Docking

**Figure 4.**
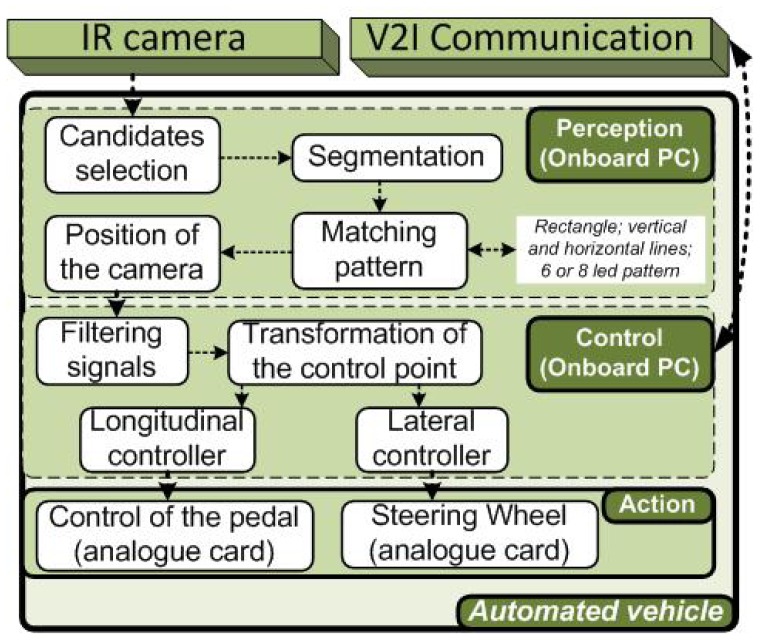
Control architecture for autonomous vehicles based on IR camera information.

Figure 4 shows the control scheme of the autonomous vehicle docking proposed in this work. It considers an infrared camera for the localization of the vehicle in the reference frame of the charging station. After pattern processing, the relative position is given to the control stage. Then, this position is filtered and translated to the center front axis of the vehicle, to improve the control accuracy. Finally, a reference command is sent to the action stage. The explanation of each module is described below.

### 3.1. Perception

A standard charge-coupled device (CCD) camera, equipped with an IR filter, was provided by our industrial partner in the project and was used in this work, placed behind the rear-view mirror, looking forward. In our experiment, infrared LEDs were used on the docking station, instead of visible beacons, to simplify their detection from the background and make the system invisible from passers-by.

The docking station is equipped with eight infrared LEDs, their positions being precisely known in the station referential. This rather high number of LEDs was chosen to allow the detection of several patterns, in case one or several lights were obstructed or failing. Our experiments showed that six LEDs were enough in practice to accurately determine the vehicle position with regard to its docking station.

Thanks to the camera information, the perception stage computes the position, in Cartesian coordinates, and the heading with respect to the reference line, then sends it to the control stage.

#### 3.1.1. Vision Detection Algorithms

**Figure 5.**
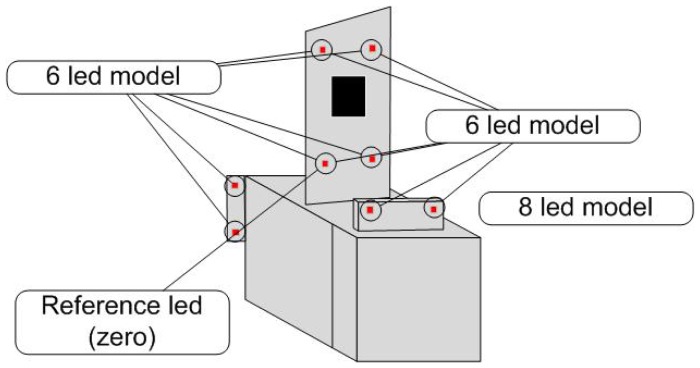
Detection algorithms using different models.

**Figure 6.**
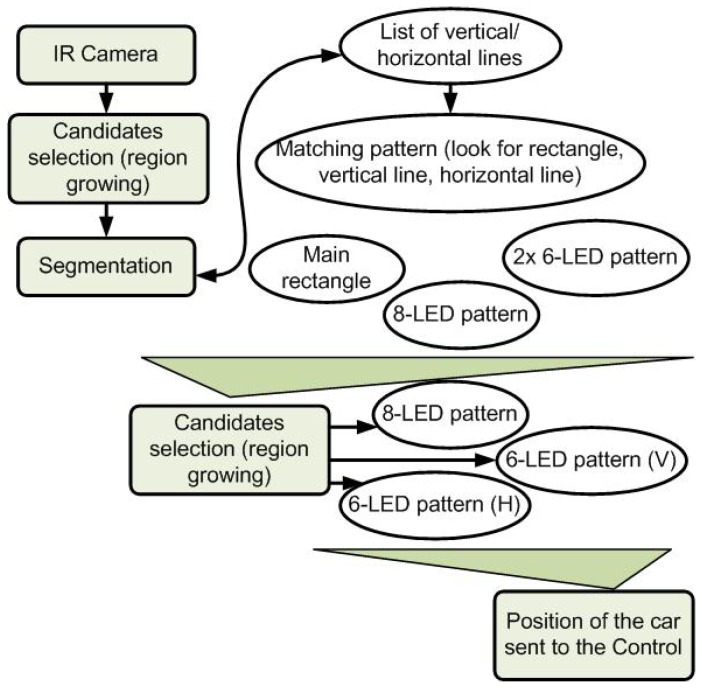
Summary of the perception pipeline.

This section describes the several steps used in the vision pipeline to get the relative position and the information needed by the control node. From the input picture, the following steps are applied:

1. *Maxima selection*. We assume that the LED candidates on the picture are among the brightest points and that they correspond to a local maximum. This is very common for the detection of bright features on a picture.
2. *Region growing*. From the previously selected extrema, region growing is applied to get the bright area. Region growing halting criteria are based on brightness gradient and absolute brightness level. The LED models being previously known, a fast model-based selection is used to remove an initial set of outliers, *i.e.*, bright areas physically too big to be our LEDs. This rejection effectively handles major light sources, such as car lights, sun light or most secular reflections.
3. *Model fitting*. A list of vertical and horizontal lines stem from the set of LED candidates previously detected. Knowing the 3D base model of the station, simple heuristics are used to remove candidates leading to an improper form-factor. In our case, several constants in the LEDs relative position (only two LEDs on top) are easy to use to remove trivial misfitting candidates. Moreover, this step can be simplified for the following detections, a rough initial position for the projected pattern being given by a previous iteration. Several LED sub-models can be tracked on the station, for an extra robustness against occlusions. In our case, three sub-models can be used while keeping the POSIT algorithm running (defined by six to eight LEDs), while a rough position can be computed from the top four LEDs (Figure 5).
4. *POSIT algorithm* (detailed in Figure 6). This algorithm, detailed in Section 3.1.2, provides an estimation of the 6D position and attitude as regards the model from its projection into the camera plane. This gives a complete determination of the car attitude. POSIT can be run separately on each of the four models detected.

#### 3.1.2. The POSIT Algorithm

This algorithm was first published in [30] by DeMenthon *et al.*, its purpose is to find the pose of an object with regard to the camera referential from a single image. This is not a simple task, due to the loss of information consecutive to the projection process from the 3D model to the picture plane. Extensive pose information typically transfers into six degrees of freedom, degrees which are not necessarily visible after the projection onto a 2D plane consecutive to the imaging process. In other words, the projection matrix stemming from the standard pinhole camera model is not invertible.

It is then compulsory to find an estimation of the pose, with an approach robust enough to handle these ambiguities gracefully. Several methods were developed over time (see the references for an extensive review), but the *POSIT* algorithm is now commonly used for this task, due to its very low coding and computing complexity and its iterative nature. *POSIT* does not require an initial pose estimate and can run in real-time on low-power hardware.

Summing up some of its key ideas, *POSIT* can be split into two steps: pose computation from an approximated scaled orthographic projection and an iteration procedure that allows the algorithm to converge without an initial guess. The scaled orthographic projection is close to a perspective projection, but differs in that the depth coordinates of the model features get the same value in the projection computation, thus neglecting intra-object depth differences compared to camera-to-object distance. This effectively linearizes the projection process. The iteration procedure consists in computing the mismatch between the observation (“true” projection of the 3D model onto the image plane) and the computed scaled orthographic projection, which gives the pose correction step.

In practice, POSIT converges within 10 iterations, and its reliability can be assessed by computing the model feature positions onto the image plane from the computed pose, camera pin-hole model and the known geometry of the model. A limit of the POSIT approximations can, however, be observed at a very close range, when the model depth dimension is not negligible compared to the camera-to-object distance.

**Figure 7.**
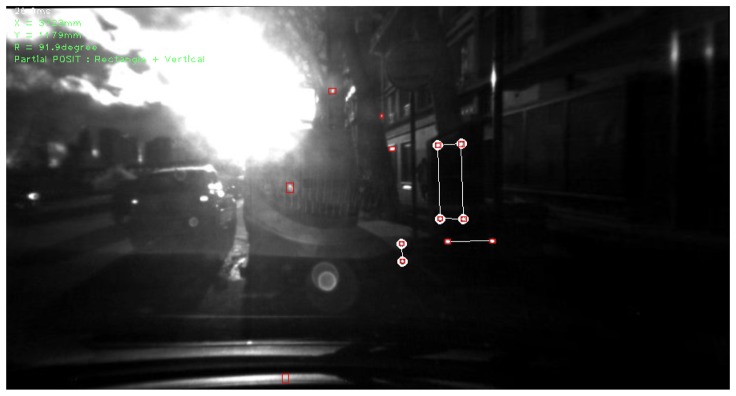
Typical view from the system, on the side of a busy road.

Figure 7 shows a typical view from the perception system. The docking station is correctly identified and positioned, as shown by the back projected features of a six-LED model (white circles) piled onto the detected LEDs (end of the white lines). This scene shows a difficult situation, because of the low position of the sun (in front of the camera), and some reflections are detected as a “worst-case scenario”. Following the proposed algorithm, an initial region growing algorithm restrained to the LEDs' reasonable size allows us to create a list of LED candidates, pictured in red squares on Figure 7. The knowledge of the positions of the LEDs relative to one another is then used to remove improbable LED configurations. The recognized configuration is depicted by the white segments in the figure. Finally, the POSIT algorithm can be applied on this recognized projected pattern, in this case on the six-LEDs sub-model, and the 3D configuration is back-projected on the picture, as shown by the white disks. The correspondence between detected LED positions and 3D projections from the known model and pose is used as quality check criteria.

#### 3.1.3. Filtering

Since the information coming from the camera signal is noisy, signal filtering is required. To this end, a digital filter implementation in terms based on classical finite impulse response and numerical differentiation is used. This technique has been developed in the framework of the project ALIEN (http://raweb.inria.fr/rapportsactivite/RA2010/alien/uidl.html), which is devoted to the study and to the development of new techniques in identification and estimation [31].

The signal coming from the camera is approximated as a truncated Taylor expansion at order *N* and for *t* = 0.

(1) $$x(t)=\sum_{i\geq 0}^{N}x^{\left(i\right)}(0)\frac{t^{i}}{i!}$$

Then, each processed signal can be extended in a polynomial function of higher degree and the derivative coefficient can be calculated by using the Laplace transform. Here, the *x*(*t*) and *y*(*t*) positions over time are locally represented as a first order polynomial function, ∀(*a*_0_, *a*_1_) ∈ ℝ^2^:

(2) $$x(t)=a_{0}+a_{1}\times t$$

In order to smooth the signal, coefficient *a*_0_ for *x*(*t*) and *y*(*t*) signals must be estimated. Using the Laplace transform and successive calculus transformation, Equation (2) can be expressed in the Laplace domain as:

(3) $$\frac{a_{0}}{s^{2}}=2s\frac{X(s)}{s^{2}}+\frac{1}{s}\frac{\text{dX}(s)}{\text{ds}}$$

where *X*(*s*) is the operational expression of *x*(*t*) (and, respectively, *Y*(*s*) with *y*(*t*)). Using classical operational to time domain transformation rules and the Cauchy formula, estimation of the coefficient *a*_0_ can be limited to one integral:

(4) $$a_{0}=\frac{2}{T^{2}}\int_{0}^{T}(2T-3\delta )x(\delta )d\delta$$

where *T* is the length of the integration window. More details on this technique are provided in [31–33].

### 3.2. Control

Different environments and conditions (speed and data available, among others) determine the control law used for autonomous vehicles. Since a real vehicle is a multipart system, some works consider complex models or AI techniques to control the vehicle [34,35]. However, under stringent conditions, such as low constant speed and the absence of dynamic forces (lateral acceleration is zero), a simple kinematic model can be used. Moreover, it is well accepted in the literature to separate the control in the lateral (steering wheel) and longitudinal (throttle and brake) for driverless vehicles, both in hardware and software. Consequently, each system can run independently.

For the longitudinal controller, we used a proportional integral (PI) to reach the reference speed, and then to reduce the speed when the vehicle is reaching the docking point. Both controllers, lateral and longitudinal, were tested in previous simulations, showing good results [29]. However, in the real implementation, only the longitudinal control worked appropriately due to the information coming from the camera, and that is always available. The bang-bang control law, proposed for the lateral control in [29], has been discarded, because the maximum vision range of the camera is limited to [−20, 15] degrees, and there is no odometry integrated in the vehicle. Moreover, in this simulation, the footpath, where the charging station is placed, was not considered; therefore, the overshoot of this control law can crash the front right wheel into the infrastructure. In this section, a new solution for the lateral control in autonomous docking for electric vehicles is presented.

#### 3.2.1. Kinematic Model

**Figure 8.**
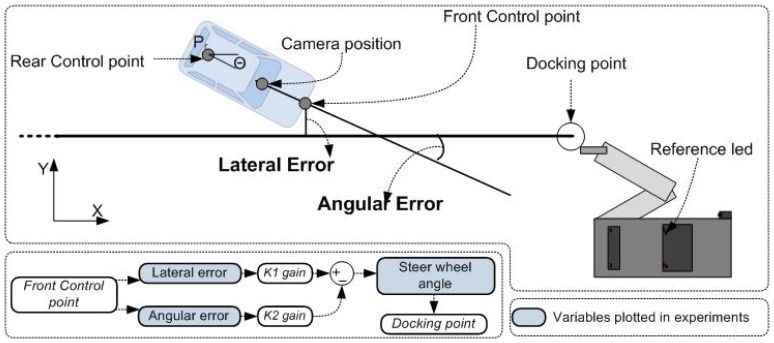
Variables used in the autonomous docking and experiments.

Due to the low speed of our application, the centrifugal force is considered as despicable; the wheel slipping and the forces transferred between wheels of the same axle track are approximated to zero. Moreover, the radius of curvature is assumed to be bigger than the wheel base. Therefore, the kinematic model is estimated by the standard bicycle or Ackerman model [36,37], considering that the two front wheels turn without different speed and the rotation center is the medium between them. The differential equations, describing the movement in a Cartesian plane (x, y), are as follow:

(5) $$\frac{\text{dX}}{\text{dt}}=V_{\left(t\right)}\times \text{cos}(\theta )$$

(6) $$\frac{\text{dY}}{\text{dt}}=V_{\left(t\right)}\times \text{sin}(\theta )$$

(7) $$\frac{d\theta }{\text{dt}}=\frac{V_{\left(t\right)}}{L}\times \text{tan}(\alpha )$$

where *θ* is the orientation angle with respect to plane XY, *α* is the steering angle of the front wheel, *L* is the wheel base and *V*_(*t*)_ is the longitudinal speed. The point X and Y are defined with respect to the center of the rear axle of the vehicle. The simulation presented in [29] shows good results controlling the rear point (Figure 8). However, due to the high precision needed in our application (the vehicle has to reach the docking point with an error of ±5 cm), it is necessary to translate the control point to the front. The bottom left part of Figure 8 shows a block diagram with the input variables used in the control stage, as well as the steering angle output, which reaches the docking point. The next module explains the considerations to this end.

#### 3.2.2. Front Control Point

Information coming from the camera provides the position (in Cartesian coordinates) and the angular error from the reference line (in radians) regarding the camera position (Figure 8). The aim of this new module is to calculate the coordinates of the control position from the coordinates of the camera, and also to fit the angle error.

Table 1 shows the properties measured from the docking point to the “reference LED” to calculate the position (Figure 8). The *Ang_offset_* is the balance of the camera with respect to the reference line, since it is slightly turned to capture more LEDs on the right side of the vehicle. The *Dist_target_* is the distance from the camera (in the rear-view mirror) to the nose of the vehicle (where the front control point is placed). The *X_offset_* and *Y_offset_* are offset distances from the reference LED to the docking point (Figure 8).

The new control points and the angular error are calculated as follows:

(8) $${\text{Angle}}_{\text{new}}={\text{Angle}}_{\text{camera}}-\frac{{\text{Ang}}_{\text{Offset}}\times \pi }{180}$$

(9) $$X_{\text{front}}=X_{\text{camera}}-\text{cos}({\text{Angle}}_{\text{new}})\times {\text{Dist}}_{\text{target}}-X_{\text{offset}}$$

(10) $$Y_{\text{front}}=Y_{\text{camera}}+\text{sin}({\text{Angle}}_{\text{new}})\times {\text{Dist}}_{\text{target}}-Y_{\text{offset}}$$

#### 3.2.3. Lateral Control

Two control variables that were used for the lateral control law are the lateral and angular errors, as proposed in previous works [37,38]. Both errors are calculated in the front control point (in meters) and the reference line (in degrees), respectively. *K*_1_ and *K*_2_ are the gains fixed manually on the vehicle. The first has a proportional effect in control action, since it is associated to the error in *Y*. Otherwise, *K*_2_ has a derivative influence in the control behavior $\frac{\text{dY}}{\text{dt}}$. From Equation (6), two facts can be assumed: the speed is constant in our experiments, and the orientation angle (*θ*) is small (constraints of the camera information). Then, Equation (6) can be rewritten as follows:

(11) $$\frac{\text{dY}}{\text{dt}}=V\times \theta$$

where *θ* is proportional to $\frac{\text{dY}}{\text{dt}}$ (angular error). Therefore, *K*_2_ has a derivative action in our system. According to the control systems controlled by a PD, *K*_1_ reduces the lateral error (meters) and *K*_2_ helps to avoid oscillations and allows a faster and softer output. The final values used—not normalized steering wheel output—are 700 and 45, respectively. Finally, an explicit form of the control law used, showing the proportional and derivative terms—according to the reference line (*Lat_error_*)—is rewritten as follows:

(12) $$U_{\left(t\right)}=K_{1}\times {\text{Lat}}_{\text{error}}-K_{2}/V\times \frac{{\text{dLat}}_{\text{error}}}{\text{dt}}$$

## 4. Results and Discussion

**Figure 9.**
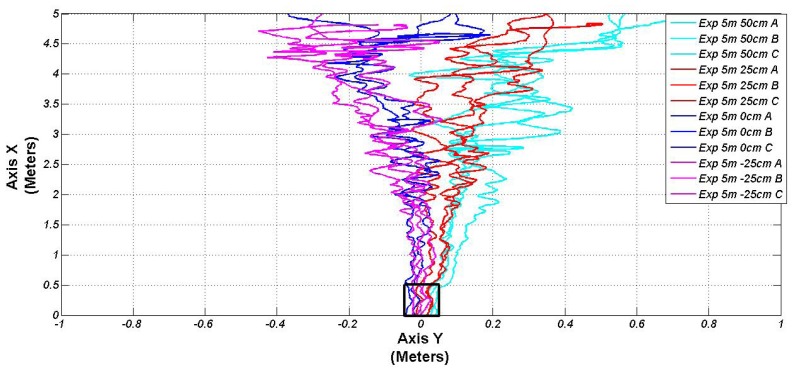
Validation tests: positioning.

After the authentication of the perception system, a validation of the entire system implemented in our electric vehicle is described (Figure 1). They illustrate the performance from different X and Y starting points (from 3 to 5 m, and 0 to 50 f, respectively). Due to the footpath, the negative values of the Y axes are not considered for real implementation. However, one experiment was completed from −25 cm to validate our control architecture. All the experiment performance in the subsection were carried out in the INRIA facilities with the same vehicle, charging station and perception system described in Section 2.1. Figure 9 shows four different validation tests. Every experiment was executed three times around the same starting reference. This figure shows the position in Cartesian coordinates, coming from the front control point module, described in Section 3.2.2. In the lower middle part of the same figure, a reference square shows that the vehicle arrives with a small error to the docking point (≤±50 mm).

**Figure 10.**
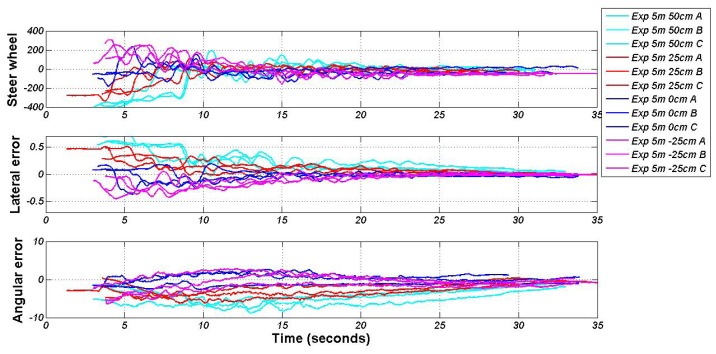
Validation tests: input variables and action lateral controller.

The upper picture in Figure 10 shows the steering wheel control output according to each experiment. The light blue graphic (departure point 5 m and 50 cm for X and Y axes, respectively) shows that the steering wheel is turning around −400 degrees, and then softly, it returns to the center. The trajectory is continuous and without overshoot due to the filtering of the input variables (Section 3.1.3). The middle and the lower pictures show the evolution of both input variables: the lateral and angular error. Both have a tendency to zero, and the error in both the lateral and angular (yaw) are small (Table 2), creating a good docking between the vehicle and infrastructure charging arm. The lateral error has been measured with an external distance measure laser in order to have real values concerning the distance between the vehicle and the docking point.

Table 2 shows the departure and arrival points in every experiment in millimeters, as well as the yaw of the vehicle. Both lateral and longitudinal controllers have reached the minimal error permitted in our application. The averages of the lateral and longitudinal errors, considering the set of experiments, are 24.7 and 9.61 millimeters, respectively. Both errors are low, and the vehicle docks inside the valid range (≤±50 mm). Moreover, the arrival yaw is also low (the root mean square error is 1.05 degrees). It is important in order to have better docking in the charging station.

**Figure 11.**
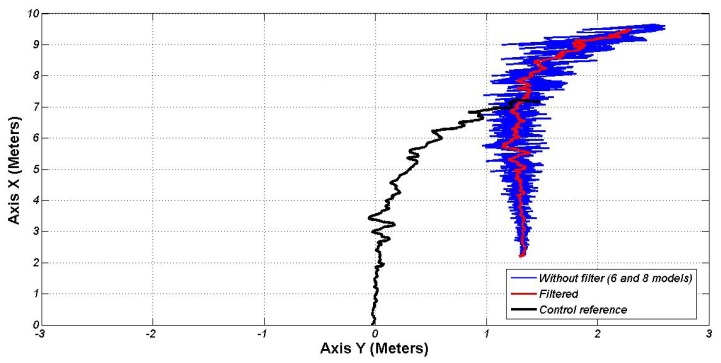
Positions given from the perception system in the final experiment.

Finally, an experiment from a greater distance, both in X and Y axes (7.5 and 1.25 m, respectively), has been performed. Figure 11 shows the position in Cartesian coordinates from the perception system, filtered and translated to the front control point. As in the previous experiments, the vehicle reaches the docking point with an error lower than 5 cm.

**Figure 12.**
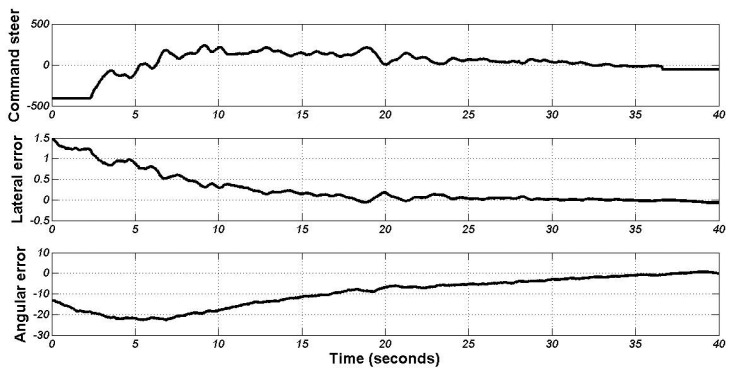
Lateral command and variables of the control in the last experiment.

Figure 12 shows the evolution of the steering position and both input variables. The control action is soft and continuous, and the vehicle never overpasses the reference line (zero in the Y axis). Around 20 s of the experiment, the vehicle arrived to the center of the docking line, but it is not completely straight; then the angular action turned the steering wheel until (35 s) the vehicle reaches the docking point with a lateral and angular error of 2 cm and 0.4 degrees, respectively. Then, the automatic charging arm is ready to charge the batteries.

## 5. Conclusions and Future Work

In this work, a control architecture for autonomous docking systems, based on an embedded perception system in an autonomous electric vehicle and a recharging station for urban parking areas, is presented. Our approach has been developed under the framework of the AMARE project, using the information provided by an infrared camera and diodes installed in the recharging station. The information from this sensor had been processed and filtered and then sent to the control stage for automatic docking of the vehicle. The proposed architecture is easily adaptable to any commercial electric vehicle.

Different experiments, departing from different points, show good behavior of the proposed system. Both lateral and longitudinal errors are lower than the limits of the charging station. The proposed controller is easy and intuitive for tuning, and the gains can be adapted according to the different vehicles' characteristics. This technology assists human drivers in the charging and docking process of electric vehicles in cities.

The system presented in this paper is actually working in a real scenario in the city of Paris (http://www.modulowatt.com/Modulowatt_video_Mobilier_Urbain_Intelligent_fr.html) as a permanent demonstrator of the AMARE project.

The proposed work relies solely on the information from the camera on board the vehicle. When the charging station is out-of-range, the camera is obstructed, the signal is too noisy or is lost (e.g., if the steering wheel turns a lot), the autonomous docking maneuver is stopped until the signal is perceived again. For this reason, other sensors and data information may be added to the control architecture proposed, such as CAN frame data or odometer data, in order to increase the redundancy and the robustness of the system in future work. Moreover, actions over the gear shift can be considered for more constrained scenarios.

## Figures and Tables

**Table 1.** Parameters to calculate the front control point.

**Parameters**	**Values**
*Dist_target_*	1.17 m
*X_offset_*	1 m
*Y_offset_*	1.36 m
*Ang_Offset_*	2.3 degrees

**Table 2.** Departure and arrival points in different situations.

	**Actual positions and; yaw**					

**Experiments**	**Departure**			**Arrival**		

	***X****_mm_*	***Y****_mm_*	***Yaw***	***X****_mm_*	***Y****_mm_*	***Yaw***
X = 5 m and Y = 50 cm	4,988.6	481.4	−2.8	27.1	26.0	−3.2

	4,963.7	479.2	0.6	37.0	−8.2	−2.1

	4,980.4	442.1	4.9	31.5	−2.9	−2.0

X = 5 m and Y = 25 cm	5,010.5	308.9	−0.2	17.5	−5.1	−1.8

	5,011.6	299.3	2.8	33.7	6.7	−1.1

	4,996.7	250.2	−1.8	40.0	−3.9	−1.9

X = 5 m and Y = 0 cm	5,018.9	−14.5	1.4	20.8	14.5	−0.6

	5,002.2	−125.2	1.1	−25.8	16.2	−0.2

	5,004.0	−24.1	2.2	37.1	28.9	1.1

X = 5 m and Y = −25 cm	5,022.0	−251.4	1.3	24.0	32.9	0.7

	5,012.9	−202.7	−2.7	17.1	21.3	−0.1

	5,015.5	−260.8	−0.9	23.4	25.3	0.3

X = 3 m and Y = 25 cm	3,026.4	191.7	0.6	40.0	−1.0	−1.4

	3,041.8	261.1	1.1	24.5	9.4	−2.2

	3,122.5	260.7	−1.1	22.8	−15.9	−2.6
